# Isolated Trapezoid Fracture in Adolescent Goalkeepers: A Scoping Review of the Literature and a Report of Two Cases

**DOI:** 10.1016/j.jhsg.2023.09.001

**Published:** 2023-10-31

**Authors:** Maya Aldeeb, Ghislain N. Aminake, Ibrahim A. Khalil, Mike Hayton, Om El Khir Ksantini, Elisabet Hagert

**Affiliations:** ∗ Department of Medical Education, Family Medicine Residency Program, Hamad Medical Corporation, Doha, Qatar; † Aspetar Orthopedic and Sport Medicine Hospital, Doha, Qatar; ‡ Department of Urology, Hamad Medical Corporation, Doha, Qatar; § Department of Orthopedic Surgery, Upper Limb Unit, Wrightington Hospital, Wrightington, UK; ‖ Department of Clinical Science and Education, Karolinska Institutet, Stockholm, Sweden

**Keywords:** Adolescents, Carpal bone, Football injury, Goalkeepers, Wrist fracture

## Abstract

**Purpose:**

Isolated trapezoid fractures are rare injuries, particularly among adolescents, constituting only 0.4% of all carpal bone fractures. This study aims to present two cases of isolated trapezoid fracture in adolescent goalkeepers and a scoping review of the literature to provide guidelines for the management of this injury.

**Methods:**

Following PRISMA-ScR guidelines, a scoping review of reported cases was conducted. Two hundred and twenty articles were found using PubMed and Google Scholar. After full-text review, a total of 30 cases from 22 articles along with our 2 cases were analyzed based on demographics, injury mechanism, method/timing of diagnosis, prognosis, and time to recovery.

**Results:**

Thirty-two reported cases of trapezoid fractures with a mean age of 26.7 years (75% male) were found, with pain as the most common presenting symptom. A majority (78%) had initial negative findings on radiography, and the diagnosis was primarily established through computed tomography (59%; n = 19) or magnetic resonance imaging (50%; n = 16). There was a substantial delay in diagnosis (mean 26 days), primarily because computed tomography/magnetic resonance imaging was frequently ordered late. The majority of cases (78%) were managed conservatively, with immobilization periods ranging from 4 to 12 weeks. The average duration for full recovery was 4.5 months, with operative management taking 7.3 months and conservative management taking 3.5 months.

**Conclusion:**

Trapezoid fractures, though rare, are often not promptly diagnosed on initial plain radiographs, leading to a potential underreporting of cases. Because of the risk of complications associated with this type of injury, clinicians should maintain a high level of vigilance and consider trapezoid fracture as a possible differential diagnosis when presented with carpal pain, swelling, or limited movement, particularly after axial load incidents. Further research and guidelines are needed to enhance our understanding and management of this uncommon injury in the future.

**Type of study/level of evidence:**

Differential diagnosis/symptom prevalence IIIb.

Football (US: soccer) is the most common group sport in the world, but, unfortunately, it is associated with a high risk of injury especially among young goalkeepers with their rapid changes in body size, composition, and hormonal release.^1^ Data about this group of athletes are scarce, but one study has reported that the most injured part of goalkeepers is the area involving the elbow, forearm, wrist, and fingers.^2^

Hand and wrist injuries are overall prevalent among athletes, accounting for approximately 9% of all sports-related injuries. Scaphoid fractures are the most common, constituting 70% of these injuries. Trapezoid fractures represent a small fraction, around 0.4%, of the total cases of carpal bone fractures, and the rarity of injury is attributed to the bone’s inherent stability with robust ligamentous attachments.^3^^,^^4^ Moreover, the complexity of the carpal bony architecture often renders plain radiographs insufficient in the diagnosis of these injuries.^5^

There is a paucity of reported cases concerning isolated trapezoid fractures in adolescents. The primary objective of this manuscript is to bridge this gap by presenting two cases of isolated trapezoid fractures in adolescent goalkeepers and providing a scoping review of the available literature. The aim is to investigate the clinical presentation, diagnostic challenges, treatment approaches, and outcomes associated with these fractures.

## Case Presentations

### Case 1

A 14-year-old right-hand dominant goalkeeper presented at our sports medicine clinic complaining of right wrist pain following an injury on the football pitch 3 days earlier. The injury was sustained when boxing the ball away after a shot to the goal. He was able to finish the game. He initially consulted at another hospital the next day after the injury where a wrist sprain was suspected, and he was given a wrist brace and nonsteroidal anti-inflammatory drugs.

On physical examination, there was minimal swelling, no gross deformity, and no joint effusion. Generalized tenderness around the radial aspect of his wrist joint and painful resisted wrist motion without signs of carpal instability were noted.

**Figure 1:**
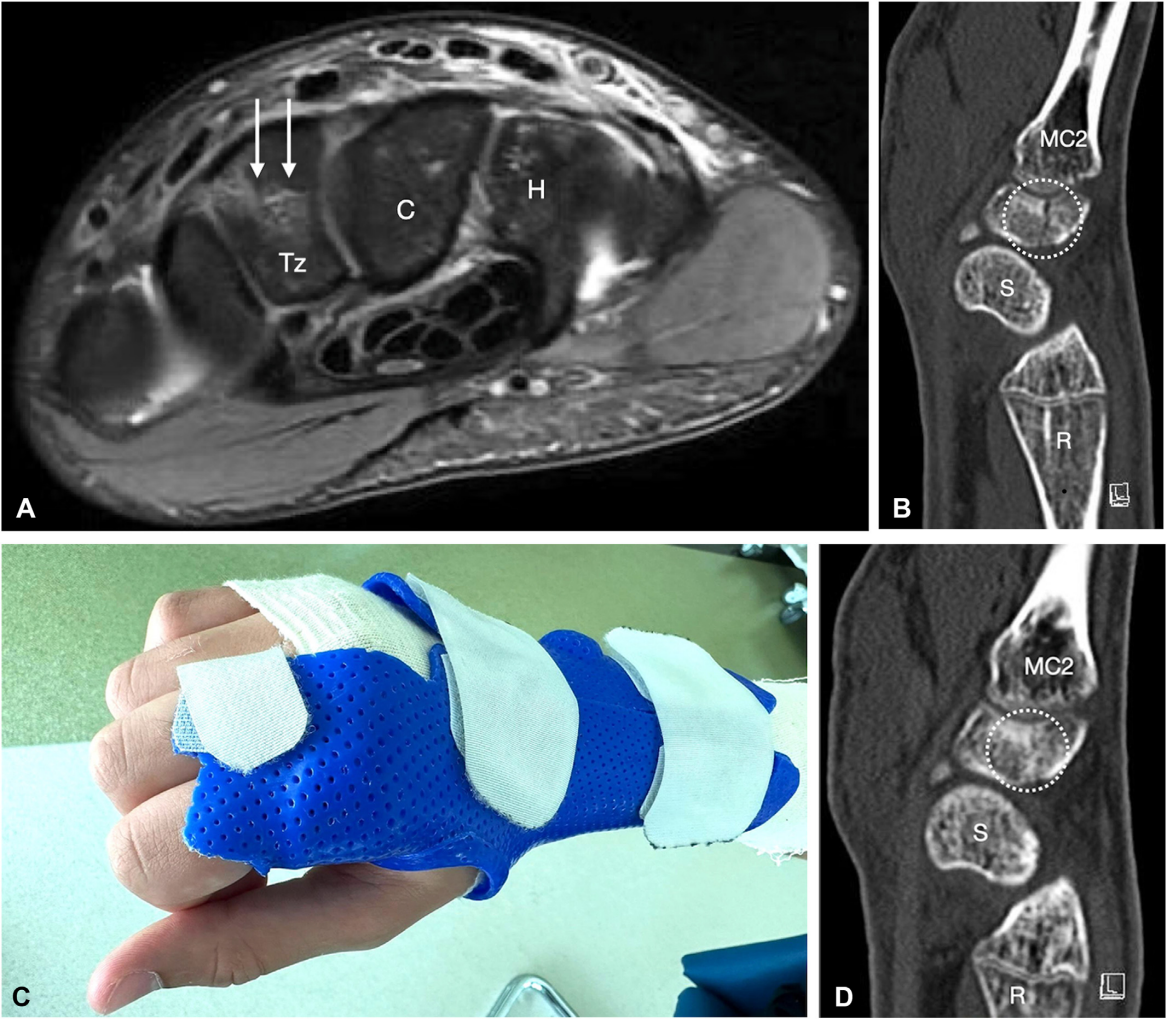
Images of the injury reported in case 1. **A** MRI T2 axial view showing a complete coronal oblique fracture of the trapezoid with surrounding bone edema (arrows). Tz = Trapezoid; C = capitate; H = hamate. **B** CT scan sagittal view after 3 weeks with visible trapezoid fracture line and questionable central bone bridging (dotted circle). MC2 = metacarpal 2; S = scaphoid; R = radius. **C** Thomine splint immobilizing the 2^nd^ and 3^rd^ metacarpophalangeal fractures and the wrist joint. **D** CT scan sagittal view with complete consolidation of the trapezoid fracture (dotted circle). MC2 = metacarpal 2; S = scaphoid; R = radius.

At this point, an x-ray and magnetic resonance imaging (MRI) were requested to rule out underlying scaphoid fracture or ligament injury. While x-rays were inconclusive, the MRI showed a complete coronal oblique fracture line in the trapezoid with surrounding bone marrow edema confirming the diagnosis of an isolated nondisplaced fracture of the trapezoid (Fig 1a). The patient was immobilized in a wrist brace, and a computed tomography (CT) scan was done at follow-up with the hand surgeon 3 weeks later, showing that the fracture line was still visible with questionable central bony bridging at the proximal aspect (Fig 1b). On physical examination, the patient still had tenderness over the fracture with worsening of pain with loading. The patient was referred to the hand therapist for a Thomine splint with immobilization of the 2nd and 3rd metacarpophalangeal (MCP) joints as well as the wrist (Fig 1c).

Three weeks later a new CT scan was done, which showed evidence of fracture consolidation (Fig 1d). Clinically, the patient had no tenderness, minimal to no pain with movements, and wrist stiffness because of immobilization. The splint was discontinued, and the patient was referred to rehabilitation for range of motion (ROM) and strengthening exercises as tolerated and was recommended to use the splint for another 2 weeks during physical load and exercises. Four months after injury, the patient was completely pain-free and has recovered full wrist ROM. His final wrist functional outcomes and PROMs are presented in Table 1.

**Table 1:** Final Functional Outcomes and PROMs of Case 1 (Conservative Treatment, 3-month Follow-up) and Case 2 (Surgical Treatment, 5-month Follow-up)

	Treatment	Functional Outcomes				PROMs∗		
		Wrist Flex/Ext	Wrist Radial/Ulnar Dev	Pro/Supi-nation	Grip Strength (Jamar, Kg)	PRWE†	Quick-DASH‡	Activity-DASH (football) §
Case 1	Conser-vative	90/75 bilat	15/55 bilat	80/90 bilat	39 injured 40 healthy	0	0	0
Case 2	Surgery	80/80 bilat	15/45 bilat	80/90 bilat	33 injured 39 healthy	0	0	0

∗ PROMS = patient-reported outcome measurements.

† PRWE = patient-related wrist evaluation (score 0-100, 0=best).

‡ DASH = Disability of the Arm Shoulder Hand (score 0-100, 0=best).

§ Activity-DASH = sports-related (0=best).

### Case 2

A 16-year-old goalkeeper presented 2 days following a fall on an outstretched left hand during a football game. The patient had a history of a prior left-hand boxing-type injury about 2 years ago during a football game. Initial x-rays at the time had revealed a comminuted trapezoid fracture that was treated at another hospital with cast immobilization for 4 weeks. The patient was then able to return to play, however, with persistent discomfort when loading.

On physical examination, he is presenting with carpal bossing at the base of the 2nd and 3rd metacarpal bones associated with significant tenderness and increased pain with manipulation.

**Figure 2:**
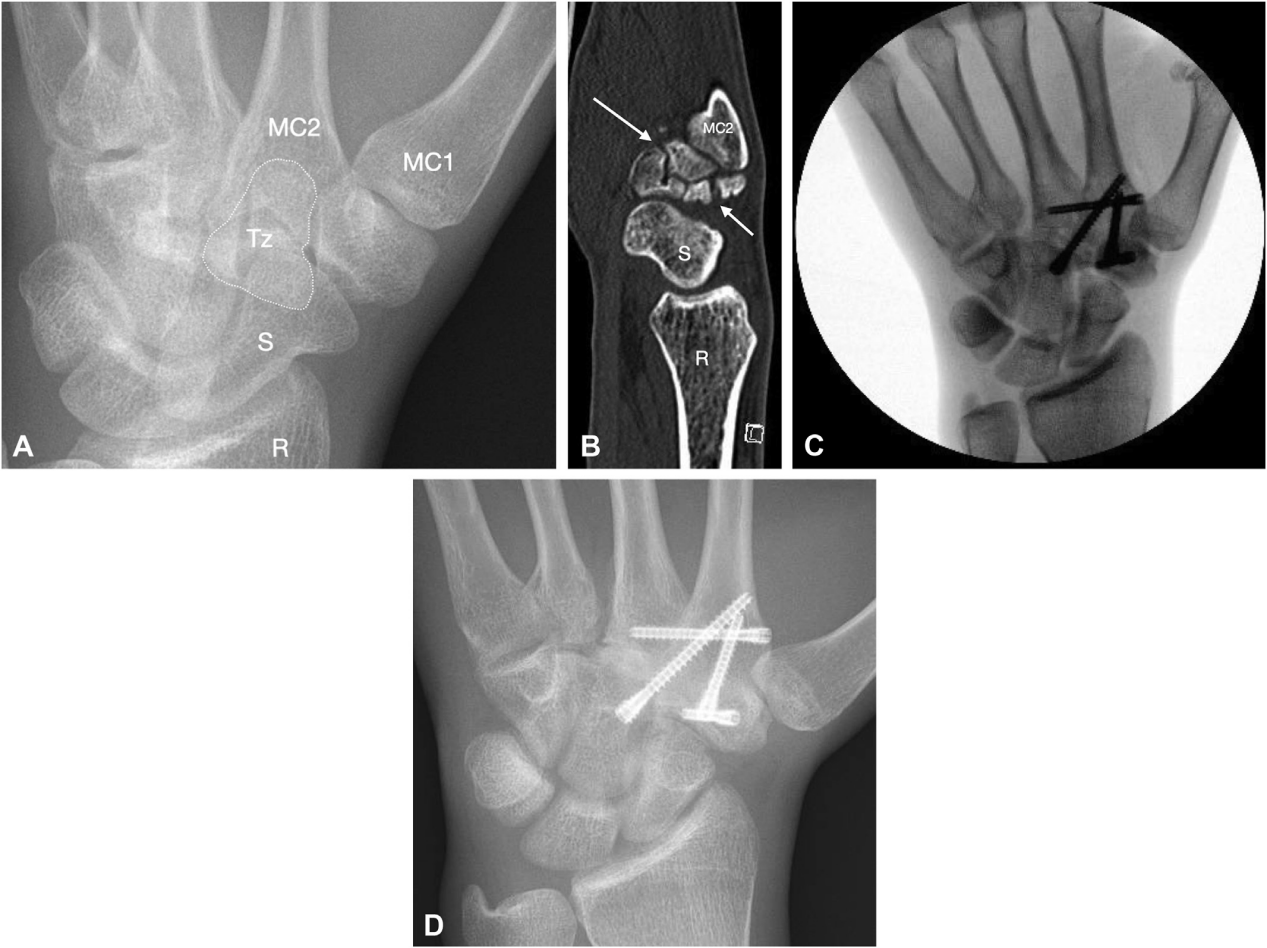
Images of the injury reported in case 2. **A** x-ray antero-posterior (AP) view view that was initially interpreted as normal, but closer inspection shows an abnormal appearance of the trapezoid (dotted line). Tz = Trapezoid; MC1 = metacarpal 1; MC2 = metacarpal 2; S = scaphoid; R = radius. **B** CT scan sagittal view showing a markedly displaced and comminuted trapezoid fracture nonunion (arrows). MC2 = metacarpal 2; S = scaphoid; R = radius. **C** Intraoperative fluoroscopy image after completed carpometacarpal (CMC) II and III arthrodesis with screw fixation and bone graft. **D** Six weeks postoperative X-ray AP view showing healed 2nd and 3rd carpometacarpal arthrodesis with screws in situ.

Plain radiographs were initially interpreted as normal (Fig 2a); however, because of clinical findings and history, additional MRI and CT scans of the wrist were done, which both revealed a markedly displaced and comminuted chronic trapezoid fracture nonunion with significant degenerative changes involving both the 2nd and 3rd carpometacarpal (CMC) joints (Fig 2b).

Owing to the chronicity of the injury and the degenerative nature of the involved joints, the patient was treated with fusion of the 2nd and 3rd CMC joints with internal fixation and autologous iliac crest bone graft under general anesthesia (Fig 2c). Postoperative management included splinting for 6 weeks after which radiographs showed healed arthrodesis with hardware in situ (Fig 2d). Clinically the patient had no pain or tenderness, good ROM, and was cleared to return to practice with a small protective wrist brace. Six months after surgery, the patient has resumed all sports activity without pain, and his final wrist functional outcomes and PROMs are seen in Table 1.

## Materials and Methods

### Case reports

The case reports are described in accordance with CARE (**CA**se **Re**ports) guidelines.

### Scoping review search strategy

The scoping review was conducted in accordance with the JBI (Joanna Briggs Institute) methodology for scoping reviews.^6^ The search strategy encompassed database searches, including Google Scholar, PubMed, as well as specific sports medicine, orthopedic, and hand and plastic surgery journals, up to June 2023. The reference lists of all included sources of evidence were screened to identify possible additional studies. Owing to the rarity of the diagnosis, all studies, including case reports, were considered. The search strategy was planned in collaboration with the institute’s librarian.

### Search terms

The combination of the keywords "trapezoid" and "fracture" was used to retrieve relevant articles. Specifically, the search query employed was "trapezoid bone [All Fields] AND ('fracture'[MeSH Terms] OR ('carpal'[All Fields] AND 'isolated fracture'[All Fields]) OR 'isolated injury'[All Fields])”. The search was limited to articles published in English and French literature.

### Eligibility

The inclusion criteria were (1) isolated trapezoid fractures; (2) clearly documented details regarding the mechanism of injury, diagnosis, and treatment options; and (3) objective or subjective measures of fracture healing and hand/wrist function as outcome indicators.

Exclusion criteria were (1) non-English or non-French articles, (2) reports involving other carpal bone and/or ligament injuries, or (3) no description of diagnostics and treatment.

### Data selection and extraction

**Figure 3:**
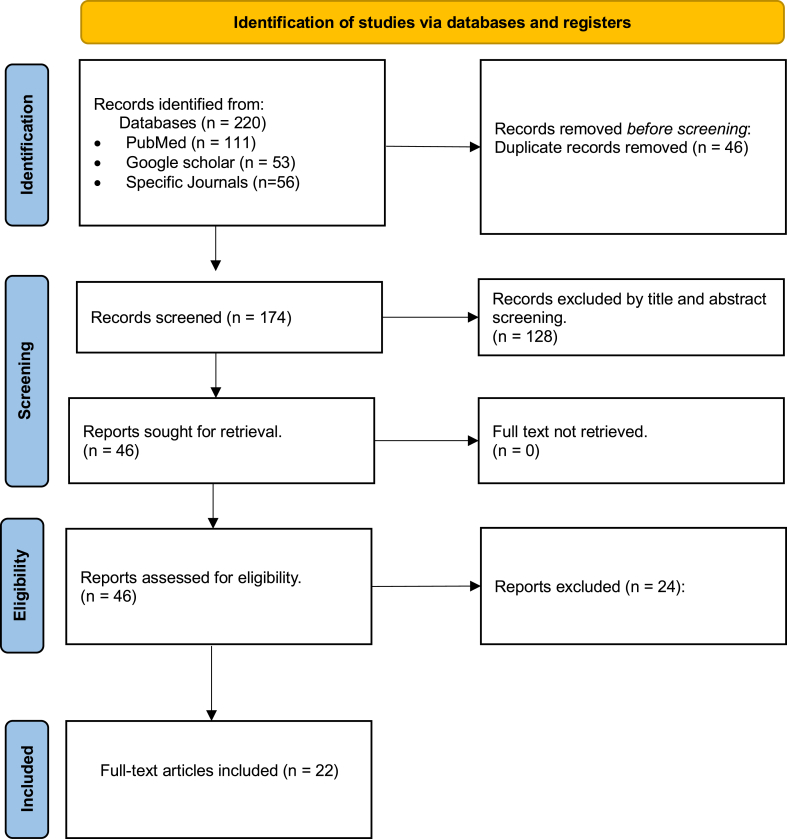
PRISMA-ScR search strategy flowchart.

The search identified 220 studies that were imported to Excel. Two independent reviewers screened titles and abstracts to eliminate 178 articles. Following full-text review, an additional 24 studies did not meet the inclusion or exclusion criteria. Data extraction was done for 22 studies, see PRISMA-ScR flowchart (Fig 3). The following parameters were extracted: patient demographics, injury mechanism, method and timing of diagnosis, prognosis, and time to full recovery.

### Quality assessment

Given the potential for bias in the case reports, the quality of each manuscript was additionally assessed using a modified version of the standardized tool developed by Murad et al^7^ (Table 2). This tool, adapted the Newcastle-Ottawa scale, comprises six questions with binary responses. A favorable evaluation necessitates a score of five points or more, a moderate assessment is indicated by four points, and a score of three or less is considered a lower quality report.

**Table 2:** Outcomes of Murad’s Tool for Methodological Qualities Assessment of Case Reports and Case Series

Author	Selection	Ascertainment		Causality	Reporting	Assessment	Quality
	1∗	2†	3‡	4§	5‖	6	
Hidlay et al^8^	No	Yes	Yes	Yes	No	No	Low
Papadakis et al^9^	Yes	Yes	Yes	Yes	Yes	Yes	Good
Nammour et al^10^	Yes	Yes	Yes	Yes	Yes	Yes	Good
Ault et al^11^	Yes	Yes	Yes	No	No	No	Low
Ribeiro et al^12^	Yes	Yes	Yes	Yes	Yes	Yes	Good
Otake et al^3^	Yes	Yes	Yes	Yes	Yes	Yes	Good
Safran et al^4^	No	No	Yes	Yes	Yes	No	Low
Afifi et al^13^	No	No	Yes	Yes	No	No	Low
Blomquist et al^14^	Yes	No	Yes	Yes	Yes	Yes	Good
Sadowski et al^15^	Yes	Yes	Yes	Yes	Yes	Yes	Good
Kam et al^16^	Yes	Yes	Yes	Yes	Yes	Yes	Good
Nijs et al^17^	Yes	Yes	Yes	No	Yes	No	Moderate
Gruson et al^18^	Yes	Yes	Yes	Yes	Yes	Yes	Good
Miyawaki et al^19^	Yes	No	Yes	No	Yes	No	low
Nagumo et al^20^	Yes	Yes	Yes	Yes	Yes	Yes	Good
Bertha et al^21^	Yes	Yes	No	Yes	Yes	No	Moderate
Heron et al^22^	Yes	Yes	Yes	Yes	Yes	No	Good
Gupta et al^23^	Yes	Yes	Yes	Yes	Yes	Yes	Good
Jacoulet et al^24^	Yes	Yes	Yes	Yes	Yes	Yes	Good
Watanabe et al^25^	Yes	Yes	Yes	Yes	Yes	Yes	Good
Bookman et al^26^	Yes	Yes	Yes	Yes	Yes	Yes	Good
Özdemir et al^27^	Yes	Yes	Yes	Yes	Yes	Yes	Good

Is the case(s) described with sufficient details to allow other investigators to replicate the research or to allow practitioners make inferences related to their own practice?

∗ Does the patient(s) represent(s) the whole case(s) of the medical center?

† Was the exposure adequately ascertained?

‡ Was the outcome adequately ascertained?

§ Were other alternative causes ruled out?

‖ Was follow-up long enough for outcomes to occur?

## Results

### Patient demographics

Thirty-two patients with a mean age of 26.7 (±10.3) years old were identified. The male-to-female ratio was 4:1.

### Injury mechanism

In terms of risk factors for high-impact hand trauma, most cases were sports injuries (n = 17), and nine of the patients were competitive athletes. Only one prior study has reported a goalkeeper-related trapezoid injury in an adult patient in addition to our 2 cases of injury in adolescents. Other causes reported are axial load because of a fistfight, a fall on an outstretched hand (n = 11), or crush injuries (n = 4). Interestingly, six patients could not recall any specific injury, and five of them presented with progressive pain. Detailed etiologies for each case can be found in Supplementary Table 1.

### Symptoms

The most common presenting symptom was pain, with 31% of cases experiencing isolated acute pain (n = 10), 15% having progressive pain (n = 5), 37.5% reporting pain and edema (n = 12), and 15.6% with pain and decreased ROM (n = 5).

### Diagnosis

During physical examination, tenderness was localized to the base of the second metacarpal dorsally in 13 cases and the radial side of the carpus in 5 cases. Fourteen cases reported tenderness on both dorsal and radial aspects of the wrist.

X-Rays failed to diagnose 78.1% of the cases (25 cases, of which 5 were displaced fractures). A majority of cases (n = 17) were diagnosed using CT, but it was falsely negative in three cases. MRI confirmed the diagnosis in 16 cases (3 of which had a normal CT scan), and only 1 case was diagnosed using ultrasound. Among the cases, 71% had nondisplaced fractures, while 29% had displaced fractures. The time between injury and diagnosis was documented in 23 cases with a mean delay in diagnosis of 26 days.

### Treatment

Conservative treatment was chosen for 78% of the cases (n = 25), involving immobilization for an average duration of 5.7 weeks using short arm splint, thumb spica, or radial gutter/sugar-tong splint. The remaining cases (n = 7) were managed surgically. Out of the 32 cases, 29 reported full recovery, while 2 cases were lost to follow-up. One case developed a nonunion because of failed initial conservative management but went on to full recovery after salvage procedure (fusion). The average duration for full recovery was 4.5 months with operative management taking 7.3 months and conservative management taking 3.5 months.

### Quality assessment

Based on the Murad et al^7^ adaptation, the quality assessment yielded good ratings for 15 studies, moderate for 2 studies, and low for 5 studies (Table 2).

### Ethics approval and consent to participate

All treatments involving the two case reports were in accordance with institutional ethical standards and the 1964 Helsinki Declaration and its later amendments or comparable ethical standards. In accordance with institutional ethical requirements (two case reports, retrospective analysis, and scoping review), this study was exempt from ethical approval. The legal guardians of the two adolescents gave written informed consent to participate and publish, according to institutional requirements.

## Discussion

Football, as the most popular sport globally, is well-known for its prevalence of football-related injuries. Injury incidences vary among players based on their position and age. However, there is a lack of data specifically focusing on injuries among adolescent goalkeepers.^1^^,^^28^^,^^29^

Recently, our institution encountered two cases of isolated trapezoid fractures in adolescent goalkeepers, which highlighted the need to review available evidence regarding the injury, diagnosis, and treatment. Owing to the scarcity of data and varying research outcomes, we undertook a scoping review to comprehensively explore the existing evidence, map the literature, and identify knowledge-gaps pertaining to isolated trapezoid fractures, especially among adolescent goalkeepers.

Some researchers have indicated that goalkeepers may experience higher injury rates in their upper limbs, particularly in their fingers, hands, and wrists, while conflicting findings have been presented by other studies.^2^^,^^29^^,^^30^ Marchessault et al^31^ mentioned that hand and wrist injuries comprise 3-9% of all sports injuries. The scaphoid is the most affected bone, especially after axial load and hyperextension trauma.^32^^,^^33^ On the other hand, trapezoid fractures, although less frequent, can occur with the same mechanism of injury. However, they are more challenging to detect as they may not be evident on routine radiographs, making them prone to being missed at initial assessment.^34^

The mechanism of trapezoid fracture typically involves high-energy injury to the hand, resulting from an axial load when the 2nd metacarpal is forcefully flexed or the wrist is hyperextended, leading to displacement of the trapezoid.^35^

The initial assessment of trapezoid fractures is challenging, as patients often present with nonspecific symptoms, such as tenderness over the radial and dorsal sides of the carpus or pain at the base of the 2nd metacarpal.^4^^,^^5^ Moreover, standard x-rays may not detect the fractures adequately because of overlapping carpal bones, resulting in normal findings in approximately 78% of cases. Delay in diagnosis is relatively common with a mean delay of around 26 days reported in our review and 17 days reported by Eckart el al.^32^

Clinicians should maintain a high index of suspicion for trapezoid fractures, especially when presented with specific indicators like mechanism of injury (boxing-type trauma, sports-related injury, and fall on outstretched hand) and physical examination findings. Early diagnosis is crucial to prevent potential complications, such as symptomatic nonunion or malunion, arthritis, or avascular necrosis (AVN).^12^^,^^21^ Surprisingly, none of the reviewed patients in our analysis developed AVN despite the trapezoid's vascularity pattern. The primary vascular supply of the trapezoid comes from the dorsal intercarpal and basal metacarpal arches along with the radial recurrent artery. However, because of the lack of internal anastomosis between the dorsal (70%) and volar (30%), any dislocations that disrupt the dorsal blood supply can place the trapezoid at risk for AVN.^31^^,^^36^^,^^37^ In the literature, only one article reported idiopathic necrosis in the trapezoid bone among adolescents.^38^

Management of trapezoid fractures varies, and no clear recommendation exists. After Safran et al^4^ reviewed 22 patients from 19 articles, they proposed a treatment algorithm for trapezoid fractures. According to their recommendations, conservative management with immobilization for approximately 8 weeks is suitable for all nondisplaced fractures and minimally displaced fractures, which they defined as < 1 mm. However, they suggested operative treatment in cases with significant displacement (> 1 mm), substantial compromise of the dorsal surface, or any breach of the trapezoidal ligaments that could potentially lead to dislocation.

In our review, we found that 78% of cases were managed conservatively with a mean duration of immobilization lasting 5.7 weeks. All patients reported full recovery following final treatment regardless of the management approach. Based on the results from the scoping review, we can recommend conservative management with a Thomine splint or cast and CT scans after 4-6 weeks to monitor fracture healing and guide the total time of immobilization. Furthermore, we agree with Safran et al^4^ that comminuted and displaced fractures should be treated surgically to avoid nonunion, as seen in our case 2.

Recovery time may differ between conservative and surgical management, with surgically managed patients requiring longer recovery periods (7.3 months), likely owing to more severe injuries. Additionally, among patients with displaced fractures, those who underwent conservative management showed a longer time to full recovery (12 months) compared to operatively managed patients (7.6 months). However, the decision should be individualized, considering the severity of the fracture and the specific needs of each patient. A case-by-case approach is essential to ensure the most appropriate and effective treatment for these rare and unique injuries.

The isolated trapezoid fractures in adolescent goalkeepers that we presented are exceptionally rare, being the two first cases described in the literature to our knowledge.^29^^,^^32^^,^^39^, ^40^, ^41^ This scarcity of cases limits the available evidence and emphasizes the need for further research to gain a better understanding of these injuries.^4^ Early diagnosis is of paramount importance. In younger patients and athletes with specific injury patterns, such as axial loading or boxing, who present with signs of focal tenderness in the radial wrist or the base of the second metacarpal, the clinician should be alerted to a possible trapezoid fracture. We recommend that these cases are treated as suspected scaphoid fractures, where clinical findings of suspected trapezoid fracture merit further imaging (CT or MRI) in case of normal initial x-rays.

While this review has provided valuable insights into the diagnosis and management of isolated trapezoid fractures, it is essential to acknowledge limitations.

Firstly, our search strategy was limited to studies published in English and French, potentially excluding relevant research published in other languages and reducing the comprehensiveness of our findings. Secondly, despite an extensive search across multiple databases, it is possible that some articles were overlooked. Additionally, our focus on peer-reviewed articles might have omitted valuable information available in nonpeer-reviewed sources, such as gray literature and conference abstracts.

Furthermore, the inclusion of only case reports and one review article in the scoping review limited our ability to draw definitive conclusions about the diagnosis and management of isolated trapezoid fractures. As a result, we focused on providing a narrative analysis of the findings.
