# Supplementary material for: Isolated Trapezoid Fracture in Adolescent Goalkeepers: A Scoping Review of the Literature and a Report of Two Cases
Source: J Hand Surg Glob Online. 2023 Oct 31;6(1):46–52. doi: 10.1016/j.jhsg.2023.09.001 (PMC10837293; doi:10.1016/j.jhsg.2023.09.001)
Supplement: Supplementary Table [file mmc1.docx]

| **auther** | **number of cases** | **age** | **Gender** | **Presentation** | **Injury** | **mechanism of injury** | **Diagnostic results** | | | **Type of Fracture** | **Time between injury and diagnosis (Days)** | **Management** | **Duration of imbolization ( Weeks )** | **Prognosis** | **Duration for full recovery ( monhts)** |
| --- | --- | --- | --- | --- | --- | --- | --- | --- | --- | --- | --- | --- | --- | --- | --- |
|  |  |  |  |  |  |  | **XR** | **CT** | **MRI** |  |  |  |  |  |  |
| Hidlay et al. ^[8]^ | 4 | 19 | M | Pain in the anatomic snuffbox, edema and decreased rang of motion | Yes | landing his fist punch during a fight | Normal | NA | Non-Displaced fracture | Non-Displaced fracture | 7 | Immbolization | Prolonged | Full recovery | 3 |
|  |  | 24 | M | Radial wrist pain | Yes | fist fight | Normal | NA | Non-Displaced fracture | Non-Displaced fracture | 35 | Immbolization | Prolonged | Full recovery | 3 |
|  |  | 18 | F | Radial wrist pain | Yes | direct trauma while playing hockey | Normal | NA | Non-Displaced fracture | Non-Displaced fracture | NA | Immbolization | Prolonged | Full recovery | 3 |
|  |  | 62 | M | Dorsal wrist pain | Yes | thrown from his motorcycle at a high speed | Normal | Normal | Non-Displaced fracture | Non-Displaced fracture | 60 | Immbolization | Prolonged | Full recovery | 3 |
| Papadakis et al. ^[9]^ | 1 | 20 | M | Pain and edema at the base of 2^nd^ metacarpal | Yes | a car door accidentally close against the back of his wrist | Normal | Displaced fracture | NA | Displaced fracture | 0 | Immbolization | 6 | Full recovery | 12 |
| Nammour et al. ^[10]^ | 2 | 27 | F | Pain and edema at radial side | Yes | high velocity motor vehicle accident | Linear fracfture | Non-Displaced fracture | NA | Non-Displaced fracture | 0 | Immbolization | 4 | Full recovery | 2.5 |
|  |  | 23 | M | Pain at the anatomic snuffbox | Yes | Baseball dynamic training caused axial force of an extended wrist | Normal | Non-Displaced fracture | Non-Displaced fracture | Non-Displaced fracture | 8 | Immbolization | 6 | Full recovery | 3 |
| Ault et al. ^[11]^ | 1 | 26 | M | Pain , edema and decreased rang of motion | Yes | hyperflexion injury after vaulting over the bicycle handlebars | Normal | NA | NA | Displaced fracture ^a^ | 6 | Immbolization | NA | Lost follow up | NA |
| Ribeiro et al. ^[12]^ | 1 | 33 | M | Pain , edema at the base of the 2^nd^ metacarpal with decreased rang of motion | Yes | heavy impact on the wrist during boxing class | Normal | Non-Displaced fracture | NA | Non-Displaced fracture | 0 | Immbolization | 4 | Full recovery | 3 |
| Otake et al. ^[3]^ | 1 | 40 | M | Pain and edema at the dorsum of the wrist | Yes | Axial load with wrist hyperextension in softball match | Coronal fracture | Displaced fracture  (6 mm gap) | NA | Displaced fracture | 3 | Operative Management | NA | Full recovery | 12 |
| Safran et al. ^[4]^ | 2 | 22 | F | Pain and edema | Yes | Crush injury | Normal | Non-Displaced fracture | NA | Non-Displaced fracture | NA | Immbolization | 6 | Full recovery | 2 |
|  |  | 46 | M | Pain | Yes | Fall skiing | Normal | Non-Displaced fracture | Non-Displaced fracture | Non-Displaced fracture | NA | Immbolization | 5 | Full recovery | 2 |
| Afifi et al. ^[13]^ | 1 | 45 | F | Pain and edema at base of 2^nd^ metacarpal | Yes | Fall | Potential fracture | Non-Displaced fracture | NA | Non-Displaced fracture | NA | Immbolization | 6 | Lost follow up | NA |
| Blomquist et al. ^[14]^ | 3 | 21 | F | Progressive pain dorsal wrist | No | Tennis player No inciting event recalled | Normal | Normal | Non-Displaced fracture | Non-Displaced fracture | NA | Immbolization | 6 | Full recovery | 2 |
|  |  | 20 | F | Progressive pain over dorsal and radial wrist | Yes | Immediate pain after axial load on extended wrist in soccer goalie | Normal | Normal | Non-Displaced fracture | Non-Displaced fracture | NA | Immbolization | 8 | Full recovery | 2 |
|  |  | 22 | M | Pain radial side and tenderness over the base of the 2^nd^ metacarpal | Yes | direct axial load with wrist extension in linebaker | Potential fracture | Displaced fracture  (1mm) | NA | Displaced fracture | 7 | Operative Management | NA | Full recovery | NA |
| Sadowski et al. ^[15]^ | 1 | 19 | M | Pain and edema at the base of the 2^nd^ metacarpal | Yes | hyperextension | Normal | Non-Displaced fracture | NA | Non-Displaced fracture | 3 | Immbolization | 8 | Full recovery | 8 |
| Kam et al. ^[16]^ | 1 | 37 | M | Pain and edema radial and dorsal aspects of the wrist | Yes | a fall onto outstretched hand ( motorcycle) | Potential fracture | Displaced fracture with bone lose | NA | Displaced fracture | NA | Operative Management | NA | Full recovery | 6 |
| Nijs et al. ^[17]^ | 2 | 30 | F | Pain at the radial side | Yes | Hit the ball with a clenched fist | Normal | NA | Non-Displaced fracture | Non-Displaced fracture | 9 | Immbolization | 8 | Full recovery | NA |
|  |  | 32 | M | Pain | Yes | Fall on outstretched hand | Normal | Non-Displaced fracture | NA | Non-Displaced fracture | 14 | Immbolization | 4 | Full recovery | 1.5 |
| Gruson et al. ^[18]^ | 1 | 25 | M | Generalized wrist pain and edema | Yes | Heavy metal door closed onto the wrist | Normal | Non-Displaced fracture | NA | Non-Displaced fracture | 1 | Immbolization | 6 | Full recovery | 3 |
| Miyawaki et al. ^[19]^ | 1 | 40 | M | Pain and edema | Yes | Axial load on a flexed wrist | Normal | Non-Displaced fracture | NA | Non-Displaced fracture | NA | Immbolization | 6 | Full recovery | NA |
| Nagumo et al. ^[20]^ | 1 | 21 | M | Pain and edema at wrist dorsum | No | Baseball player, No injury recalled | Normal | NA | Displaced fracture | Displaced fracture | NA | Operative Management (excision of bone fragment) | NA | Full recovery | 2 |
| Bertha et al. ^[21]^ | 1 | 23 | M | Progressive pain near base of the 2^nd^ metacarpal | No | Gymnast after increasing intensity of training | Normal | NA | Non-Displaced fracture | Non-Displaced fracture | NA | Immbolization | 4 | Full recovery | 3 |
| Heron et al. ^[22]^ | 1 | 22 | M | Progressive pain at the base of 1^st^ and 2^nd^ metacarpal | No | Shot-putter, No injury recalled | Normal | Non-Displaced fracture | NA | Non-Displaced fracture | 720 | Immbolization | 8 | Full recovery | NA |
| Gupta et al. ^[23]^ | 1 | 23 | M | Radial wrist pain, edema and decreased rang of motion | Yes | Hitting a ball with clenched fist | Normal | Non-Displaced fracture | NA | Non-Displaced fracture | 0 | Immbolization | NA | Full recovery | NA |
| Jacoulet et al. ^[24]^ | 1 | 20 | M | Pain | No | Swimming injury poorly defined | Normal | Non-Displaced fracture | Non-Displaced fracture | Non-Displaced fracture | 0 | Immbolization | 8 | Full recovery | 2 |
| Watanabe et al. ^[25]^ | 1 | 29 | M | Pain at the base of 2^nd^ metacarpal | Yes | Car accident | Displaced fracture | Displaced fracture | NA | Displaced fracture | 56 | Operative Management | NA | Full recovery | 6 |
| Bookman et al. ^[26]^ | 1 | 18 | M | Progressive pain of the wrist dorsal radial aspects | No | No trauma recalled | Normal | NA | Non-Displaced fracture | Non-Displaced fracture | 42 | Immbolization | 3 | Full recovery | 3 |
| Özdemir et al. ^[27]^ | 1 | 20 | F | Progressive pain over the base of the 2^nd^ metacarpal with limited range of motion | Yes | Fall on outstretched hand | Potential fracture | NA | Displaced fracture | Displaced fracture | 180 | Operative Management | 1 | Full recovery | 12 |
| Aldeeb et al.^b^ | 2 | 14 | M | Pain and edema of the radial aspect of the wrist | Yes | Boxing punch of football | Normal | NA | Non-Displaced fracture | Non-Displaced fracture | 3 | Immbolization | 3 | Full recovery | 4 |
|  |  | 16 | M | Pain, edema and decreased rang of motion | Yes | Boxing punch of football | Degenerative changes CMC 2&3 no fracture noted | Displaced fracture | Displaced fracture | Displaced fracture | 2 | Operative Management | 6 | Full recovery | 6 |

**Table 3: Demographic and clinical details of the cases found in the scoping review, XR: X-ray , CT Computed topography , MRI : Magnetic resonance imaging .**

**^a^ Displaced fracture diagnosed by ultrasound**

**^b^ Summary of our cases presented in this report**
